# Nursing Section

**Published:** 1904-01-09

**Authors:** 


### The Hospital.

### Nursing Section.

Contributions for this Section of "The Hospital" should be addressed to the Editor, "The Hospital" Nursing Section, 28 & 29 Southampton Street, Strand, London, W.C.

No. 902.—Vol. XXXV. *SATURDAY, JANUARY* 9, 1904.

### Notes on News from the Nursing World.

### THE NEW QUEEN'S NURSES.

The official list of the new Queen's nurses, whose appointment Queen Alexandra has approved, appears in another column. Of these 80 are to serve in England, 7 in Wales, 20 in Scotland, and 12 in Ireland. The figures show an increase in England over last year of 16, in Wales of 2, in Scotland of 4, and in Ireland of 1. Nine of the nurses appointed in England received their district training in Bloomsbury, 10 in Liverpool, and 6 in Manchester; 18 of the number appointed in Scotland were trained in Edinburgh; and 11 of the number appointed in Ireland were trained in Dublin.

### BELATED HONOURS FOR LADYSMITH HEROINES.

At the beginning of last month in an official document headed "Further Honours for the South African War," which proved to be an addendum to a despatch from Lord Roberts dated April 2, 1901, the names mentioned by the Commander-in-Chief included those of "Nurse J. Bradbury and Nurse C. Addison of the Natal Volunteer Medical Corps." This was extremely vague, and we have made inquiries as to the identity of the nurses thus singled out for honour, and their claims to distinction. We are now able to state that Nursing Sisters J. Bradbury, who belongs to a well known family in Natal, and Constance Addison, daughter of a retired doctor in Durban, were two of the twelve trained female nurses who were chosen from many volunteer candidates and attached to the Natal Volunteer Medical Corps, the medical organisation maintained by the Natal Government in connection with the local forces of that Colony at the time of the war. Miss Bradbury and Miss Addison were also the two first nurses who when the siege of Ladysmith took place offered to stay in the town and brave the dangers of the siege, while the hospital staff itself moved out to Intombi, a place of safety. Both of them rendered splendid service throughout that most trying time, and by their brightness in all circumstances did much to keep up the spirits of the troops. Great surprise was expressed in Natal that the most valuable work they did appeared to have been unnoticed by the authorities, and the publication of the belated despatch has explained what had hitherto seemed inexplicable. The Natal Volunteer Medical Corps have long since recognised the "meritorious services performed" not only by Miss Bradbury and Miss Addison, but also by the whole of their twelve nurses, to each of whom they have presented a handsome gold watch.

### A VACANT POST AT SYDNEY.

The post of matron of the Royal Prince Alfred Hospital, Sydney, which, when complete, will be both the largest and the most modern hospital in Australia, is vacant, and English nurses are invited to apply for the appointment. Applicants should be under 35, and should, of course, have had experience in the management of the nursing staff of a large hospital, and in housekeeping. The engagement is for three years, and passage out will be paid. Candidates are asked to state salary required, but before doing this, they should write to the Agent-General for New South Wales, Victoria Street, Westminster, S.W., for a copy of the rules of the hospital. The applications, which will be received up to February 28th, will be dealt with by a committee in London.

### STATIONARY SISTERS.

There is always a danger in hospital management of assimilating the rules of the institution to the law of the Medes and Persians, and now and again it is necessary to emphasise the fact that alterations are not only possible but may be most desirable. At the Royal Prince Alfred Hospital, Sydney, the sisters are moved from their wards every six months. The system seems to have met with so much success that we think the idea of trying it here is worthy of ventilation at the beginning of a new year, and that it would be interesting to know the opinions of matrons and of sisters themselves respecting some of the following points: What are the advantages of sisters being allowed to remain in one ward for a long period of years, varying from 13 to 20? Is it not the case that if a sister is moved, she necessarily picks up hints from every one she follows, and is more likely to be put upon her mettle to leave the ward as perfect as possible for her successor? What force is there in the contention that a constant education in all the different branches of education must be better for sisters than a stationary position? Do the nurses under her gain more, or lose more, when the stationary system is in operation? Are doctors and matrons opposed to change, and if so, why? These are questions which appear to us to merit attentive consideration, and answers based upon personal experience cannot fail to be illuminating.

### DISPENSING WITH THE MATRON'S SIGNATURE.

The Infirmary Committee of the Croydon Board of Guardians reported on Tuesday that they had received a letter from Miss Vickery, late probationer nurse at the Infirmary, inquiring why her certificate of training did not bear the matron's signature. Dr. Wilson reported that he had informed Miss Vickery that she must have been well aware that it had been decided that the certificate she would receive at the expiration of her training would not bear the matron's signature. The clerk reported that he had written to Miss Vickery, stating that at the time of her appointment as a probationer nurse there was no regulation in force to the effect mentioned but he would, however, lay her letter before the next meeting of the Infirmary Visiting Committee. The committee did not recommend any alteration in the existing arrangement. The Board approved this report and adopted it unanimously.

### POLITICS AND NURSING.

The introduction of politics into nursing is hardly less to be deplored than that of sectarian bigotry. We therefore notice with regret that at a meeting of the guardians of the Birr Union, in Leinster, a member of the board, in eulogising the services of the retiring matron of the fever hospital, took occasion to refer to the lately deceased husband of that lady, and to confess his satisfaction that Mr. Francis Dooley was, like himself, "even an unflinching Nationalist." What this fact had to do with the services rendered to the patients in the fever hospital by Mrs. Dooley was not explained, and it is a pity that the guardian making mention of it was not sharply called to order. If the expression of political sentiments by persons appointing nurses for the sick is to be allowed, we shall next have nurses themselves taking sides in politics, and we know of nothing, except indifferent personal character, so certain to lower the respect at present entertained for the profession as any tendency of this kind.

### THE NEW MATRON OF THE CANCER HOSPITAL.

Miss Shotter, the new matron of the Cancer Hospital, Brompton, has been presented on her retirement from the post of matron of the Royal Infirmary, Sheffield, with several substantial tokens of regard. The resident medical staff have given her an iron and copper fire screen; the nursing staff a pair of silver candelabra; the housekeeper a silver card-tray; the chaplain and domestic staff a silver sugar basin and sifter; the laundry staff a silver-mounted biscuit box, a silver jam spoon and pickle fork; the house and sewing maids a silver-mounted toilet set; the kitchen staff a looking-glass, silver-mounted flower vases and inkstand; and the lady visitors silver-mounted flower vases.

### THE CONGRESS AT BERLIN.

For the benefit of English nurses who will either in ordinary circumstances have their holiday in June, or who can arrange to do so if necessary, we call attention to the fact that a Nursing Congress in connection with the International Council of Nurses will be held in Berlin during that month. It is stated that many American nurses have arranged to be present, and there will doubtless be a considerable European contingent. The proceedings themselves can hardly fail to be interesting and enlightening; and it will be easy to combine a good deal of pleasure with the profit which should be obtainable where a number of members of the nursing profession from all parts of the world assemble together.

### HONGKONG NURSING INSTITUTION.

At the third annual meeting of the Hongkong Nursing Institution, which was held on November 20th, the chairman expressed his belief that the financial position was not quite so satisfactory as it was the previous year, the balance of receipts over expenditure being only 140 dollars. He explained that this was due to the earnings of the nurses being rather less, to the nurses receiving some increase in salary each year they remain in Hongkong, and to the fact that owing to the lowness in exchange it has cost the committee considerably more to defray their salaries, which are paid in sterling. In 1904, he added, the agreements of the two nurses terminate, and as they have both decided to return to England the Association will have to pay half their passages home and also the expense of two outward passages for two nurses to replace them. The agreement with the nurses was that, if they stay five years, they shall have their whole passage money home, and if they remain only three years half of it. The desire of the committee is, it appears, to set aside about 600 dollars annually for passages, but in order to be able to do this they require a larger number of subscribers. At present there are only 45, and there is much force in the contention that there must be many more persons in the colony than these who are directly interested in keeping the institution from gradually eating away its guarantee fund—from which money is now borrowed—and thus coming to an untimely end. That the services of trained private nurses are appreciated in Hongkong is clear from the statement that during the last 12 months they were engaged respectively 265 and 260 days.

### BRITISH GYNÆCOLOGICAL SOCIETY.

The next examination of the British Gynæcological Society for its certificates in maternity nursing, and in gynæcological nursing will be held during the first week in March. The standard required during this year by the society is a minimum of two years' general hospital training, together with special training either in monthly nursing or in the gynæcological wards of a general or special hospital.

### THE NURSES OF ST. MARY'S HOSPITAL.

On New Year's Eve an entertainment took place in the out-patient department of St. Mary's Hospital, Paddington, for the patients and night-nurses and their friends, and on New Year's Day for the day nurses and their friends. Every nurse was allowed to bring one friend. An excellent representation of "Dandy Dick" was given, and after refreshments had been served, the Leoni Ladies' Quintette String Band performed. These reunions are always welcomed if only because they afford an opportunity for those who seldom see each other to meet and exchange greetings. Old students of St. Mary's, now flourishing practitioners, and former nurses, some of them now matrons, were very much in evidence on Thursday and Friday last week.

### A SISTER MARRIES A PORTER.

Just before the end of last year, Miss Lynch Blosse, one of the sisters at Cardiff Infirmary, left the institution. She had been probationer, staff nurse, and sister in succession, and her services were much valued. The daughter of a dean, she possessed an income of her own, and always returned her salary to the governors in the form of a subscription. She also collected considerable sums of money from her friends for the benefit of the infirmary. For every reason, therefore, her resignation was regretted, and this regret was not diminished when it became known the other day that Sister Blosse had privately married the porter before she severed her connection with the infirmary. The porter had only been in the employment of the governors a short time, but he is described as a well-set up man, who went through the South African war.

### NURSES AS STEWARDESSES.

As we continue to receive letters from nurses who desire to take up the duties of stewardess on an ocean-going vessel we cannot do better than refer them back to the communications of the two correspondents which appeared in our issue of Dec. 20. Both of these have had practical experience, and it is significant that they arrive at an entirely different conclusion. One is clearly of opinion that, providing a nurse be physically robust, the post of a stewardess, if it can be obtained, is far from unpleasant. The other, whose experience is very recent, shows that under the auspices of one company, at any rate, hospital-trained nurses who answer advertisements for "lady stewardesses" must not expect to be treated as ladies when they get to sea. The latter naturally recommends nurses who are tempted to travel in the same capacity to ascertain, before they agree to start, the precise standing they will occupy on board.

### A WARDSWOMAN AS NIGHT NURSE.

The Wycombe Guardians have decided, after a discussion of considerable length, to appoint a wardswoman to take charge of cases in the workhouse infirmary during the night. This is the result of statements by the medical officer that the two nurses hitherto forming the staff were outrageously overworked. The nurses themselves were summoned before the Board, and in reply to questions said that they had found the work much heavier than they expected it would be. They seldom got to bed before 12 o'clock and were liable to be awakened at any hour during the night. In spite of such conclusive evidence of the need of assistance, seven Guardians voted against any help being given. It is a pity that the majority of 16, instead of resolving to engage a wardswoman, did not determine to appoint a third nurse. According to the medical officer there are 30 old people in the infirmary who are quite helpless from great age and infirmity. Yet these are to be looked after at night by an entirely untrained person. If the overworked nurses gain by the new arrangement the poor patients are likely to suffer from it.

### A PROBATIONER TREATING A PATIENT.

The Birkenhead Board of Guardians have had under discussion a case of alleged neglect in the nursing of the sick at the workhouse infirmary. The fact that the Local Government Board inspector, while calling the attention of the medical officer and the superintendent nurse to the case and expressing the hope that there would be no occasion for such complaints in future, did not make a formal report, was cited by members of the Board as proving that he did not consider it of much importance. But it transpired that a probationer had been treating a patient on her own responsibility, and that, although the treatment was entered in the ward book, the entry had not been noticed by the superior officers. We have only to say that in a well-ordered institution such a state of things as this would not be possible.

### BRUTALITY BY A DRUNKEN NURSE.

On Saturday the magistrates at the Bullingdon Sessions, held at Oxford, sentenced a nurse at the Thame Workhouse to six weeks' imprisonment, with hard labour, for a shameful assault upon an aged female inmate of the sick ward, who was suffering from paralysis. The evidence against the accused was conclusive The master of the workhouse proceeded to the ward at half-past twelve at night because he heard screaming proceeding from it, and he arrived in time to see Nurse Jones beating the patient with a stick; while the medical officer, who was called to the workhouse at one in the morning, found that the woman had severe injuries on the face and head, and marks on one arm. Both witnesses said that the nurse was in a drunken condition. This, however, was only an exaggeration of her offence, and it may be hoped that a just conviction and a well-merited punishment have terminated the career of a person who has proved herself so utterly unfit to discharge any of the duties of a nurse.

### A QUEEN'S NURSE IN AN IMPOSSIBLE POSITION.

An attempt is now being made to carry on the work of the Bishop Auckland District Nursing Association by combining members of the present committee and members of the committee who resigned in 1902. If, as we understand, a Queen's nurse is to be employed to attend other patients than the sick poor, the managers will be departing from the fundamental principles of Queen Victoria's Jubilee Institute. On the other hand, as the working men in the town are apparently quite satisfied with the organisation which was founded when the original association adopted the lines of a provident institution, we do not see how it can be reasonably expected that they will support a second.

### NEW NURSING ASSOCIATIONS.

At Montgomery and Ashington, in Sussex, and at Blofield, in Berkshire, it has been decided to form nursing organisations. The Montgomery people owe much to the ex-Mayor, who has offered to bear a third of the cost of a trained nurse. At Ashington, which is to be the centre of a considerable district, it appears to have been decided that an income of £50 a year will suffice, but this sum will not adequately provide for the services of a trained nurse. They know better at Blofield, where it is proposed to start a nursing guild with £150, and to secure a Queen's nurse. At Carleton Rode, in Norfolk, we regret to say, the nursing association established five years ago for the use of Carleton and Bunwell, has been disbanded owing to lack of financial support.

### NURSE AND NURSEMAID.

Last week the daily papers published a report that "Emily Bailey, a nurse, of Tunbridge Wells, was watching a workman destroy a tree when a portion of it fell and killed her." The sad incident occurred, but the unfortunate victim was not a nurse. She was a nursemaid of seventeen in the service of one of the masters of Clifton College. Even the local prints publishing full details of the disaster headed their reports, "Fatal Accident to a Nurse."

### SHORT ITEMS.

Miss P. Young and Miss E. T. Noble have resigned their appointments as sisters, and Miss L. F. A. Waller, her appointment as staff nurse in Queen Alexandra's Imperial Military Nursing Service.

### The Nursing Outlook.

"From magnanimity, all fear above;
From nobler recompense, above applause,
Which owes to man's short outlook all its charm."

### RIVAL REGISTRATION BILLS.

A DRAFT bill for the registration of trained nurses is being circulated amongst certain hospitals and nursing institutions just now, with a request for remarks and suggestions to be sent to the Society for the State Registration of Nurses. The bill begins by saying that it is "expedient" that persons requiring nursing assistance should be enabled to distinguish qualified from unqualified nurses, and that therefore a General Nursing Council should be established, whose duties should be to register certain nurses and to "regulate, supervise and restrict within due limits the practice of trained nurses," and to "issue and cancel certificates."

We have often stated our view that it would not enable the public to distinguish between the qualified and the unqualified nurse to have registration: we say that even now a certificate is often valueless and that knowledge crammed for an examination melts quickly away, and that the best qualities of a nurse are not registerable. What is equally wanted in a nurse with theoretical training is heart wisdom, and the gentle hand and observant eyes are as important as pharmacy and therapeutics put together. We think everyone of common sense will agree that here no Act of Parliament can help us, but rather hinder us. Secondly, there is the constitution of the proposed General Council to be considered, for it will be seen that their powers are great over those who bow down to them and consent to go on their proposed register. The vast majority of the Council are to consist of "registered" nurses, and the President is to be a "registered" nurse, and there are to be three representatives of the Matron's Council! This is laughable, and a delightful example of the complacent self-absorption of a certain few. That army and navy nursing, that mental nursing, that the Midwives' Institute and the Poor-law nurses are utterly forgotten, is typical. Of course Parliament would never even consider such a faulty scheme, so obviously drawn up from mere personal glorification and ambition. If good work is to be done it must be done on a broad and unselfish basis; its aim must be to protect the public as a whole, to help the whole nursing body; and all sectional barriers and petty tyrannies must be made impossible. It is only too evident what would be the fate of the majority of nurses if they put themselves in the power of such a petty-minded posse as this. The woman who nurses the mentally ill is often as well trained and frequently more worthy and more hard-worked than her sister who cares for the physically ill. Why, therefore, are asylum nurses to have no consideration, no place, no representative? The Poor-law nurse (whose status and prospects we have discussed so lately that we need not go into them again) is important enough and numerous enough to demand to be heard and respected. They would all have to help to find the funds for the proposed Council's doings, and they would more than probably be called on to pay many of the Council's penalties since they would have no representative to take their part or state their case. No; so long as the proposed registration is left in these hands all nurses and all nursing authorities should fight it with every weapon in their power. This bill can find no approval we are sure, save in that funny little body which calls itself so grandiloquently and misleadingly "The Matrons' Council of Great Britain and Ireland."

Then there is a second draft-bill in circulation issued by the special committee of the Royal British Nurses' Association, to discuss which a special meeting is to be held on January 8th, at 5 P.M. It need scarcely be said, so pitifully public are the petty quarrels of the nursing world, that in this bill there is no mention of the Matrons' Council, and no confining of a majority to registered nurses. It proposed a Central Board of wider feeling:—Nine medical practitioners, three lay members, three nurse-representatives, nine matrons, and six fully-trained nurses. It admits the existence of the army and the navy nurse and the Midwives' Institute, but it also forgets the mental nurse and the Poor-law nurse. Now we get the following figures from the summary tables of the last census:—

*Inmates of Institutions in England and Wales.*

| | |
|---|---|
| Hospitals ... ... ... ... ... ... | 39,184 |
| Lunatic Asylums ... ... ... ... ... | 84,118 |
| Workhouses (including Infirmaries) ... ... | 208,650 |

by which it will be seen that the mentally ill and the number of paupers are far greater than the number of persons in hospital. Of the number of private patients we cannot get exact figures, but most certainly to send any Act to the Houses of Parliament which ignored the special claims for protection of the paupers and the lunatics, and the special needs for help of the mental nurses and the poor-law nurses is a folly of which no big-minded association could be guilty. In other ways this second bill is less objectionable; it has remembered British dominions beyond the seas; it asks that all private nursing homes shall be registered and inspected; it suggests an authorised badge and keeps the mention of penalties in the background. But in spite of rules and regulations we agree with Miss Nightingale that as no scheme can secure those personal qualities without which no amount of professional skill will render a nurse a desirable attendant on the sick or inmate of our homes, registration if not mischievous is quite valueless.

### Lectures on Ophthalmic Nursing.

By A. S. COBBLEDICK, M.D., B.S.Lond., Senior Clinical Assistant and late House-Surgeon and Registrar to the Royal Eye Hospital.

### LECTURE XXVI.—DISEASES OF THE LACHRYMAL APPARATUS.

A FEW remarks must be made on this subject, as it is an important one, both from the patient's and surgeon's point of view.

The surgeon courts disaster who fails to examine the lachrymal apparatus before opening the anterior chamber of the eye, for any septicity, which is not necessarily always apparent, will certainly gain entrance to the interior of the eye, and be the means of spoiling the result of the operation and possibly of losing the eye. The most common affection is some obstruction to the normal flow of tears from the conjunctival sac to the nose, with the result that the tears run over the face; this is termed epiphora.

The causes of this affection are numerous; it may be at the orifices of the canaliculi. *The punctum*, which is normally always in contact with the conjunctiva, if the lid is drawn away from the eye (*Ectropion*) is unable to perform its function. Epiphora results if the punctum is partially or wholly occluded as the result of attacks of inflammation; if the canaliculi are stenosed, if there is a stricture of the nasal duct. These different causes of epiphora may be all more or less present in one case, so that the true condition of affairs is only discovered some time after treatment has been started.

The slight cases of epiphora are mostly due to a swelling of the mucous membrane lining some part of the lachrymal track, caused by an extension of a similar process from the nose or conjunctival sac. Such cases can be cured by treating the nose or conjunctiva and by syringing the lachrymal passages with an a stringent lotion, *e.g.* zinci chloridi gr. ii. ad. ℥i.

When the punctum is partially or wholly occluded or when there is a stricture in the canaliculus, the operation of slitting up the canaliculus must be performed. For this purpose a Weber knife is necessary; it is probe pointed. The operator stands behind the patient and holds the head firmly against himself, so that any sudden movement on the part of the patient is impossible. The edge of the lower lid is rendered tense and the punctum is drawn from the conjunctiva by drawing the loose skin of the lower lid towards the external canthus. After the probe point is in the punctum, the handle of the blade is tilted until it is horizontal and with a steady pressure the knife is forced towards the nose, care being taken that the cutting edge is directed towards the eyeball.

When the point comes in contact with the side of the nose it is in the lachrymal sac: by again raising the handle to the vertical the canaliculus can be slit as far as is considered advisable.

When the punctum only is occluded it is well to see what result is obtained by dilating it with a Nettleship probe once or twice a week.

The after treatment consists in keeping the parts clean with boracic acid lotion.

Epiphora may be complicated by the presence of a mucous or muco-purulent discharge from the lachrymal sac, with or without a swelling in the region of the internal canthus; this nearly always means that there is a stricture in the nasal duct, *i.e.* between the sac and the nose; this condition of affairs is termed *blenorrhœa*, and the swelling is termed a *mucocele* (this must not be confused with a mucocele of the frontal sinus, which is always situated at a higher level).

This mucoid discharge is caused by micro-organisms being washed into the sac by the tears, and remaining stagnant there through closure of the duct by the stricture; they find a suitable medium for multiplication and may set up inflammatory trouble.

When inflammatory trouble does arise it spreads outside the duct into the subcutaneous tissues and produces a cellulitis (*acute dacryo-cystitis*). This is accompanied by swelling, redness, and very acute pain. If untreated, the cellulitis may spread to the lids and completely close them. The treatment consists in free purgation and the application of hot fomentations to the affected part. When fluc tuation is definite, an incision should be made through the skin over the most prominent part of the swelling, and the abscess freely opened. If the opening is free no drainage is necessary, but fomentations should be continued until the surrounding swelling and redness subsides. Subsequently the stricture must be treated.

These cases of stricture of the nasal duct are most troublesome. The canaliculus must be slit, as previously described, and the stricture dilated with probes. The smallest probe should be passed carefully, so as to avoid making a false passage into the nose; when the stricture is once passed, larger probes can be introduced without difficulty and the duct so restored to its normal calibre.

The tendency is for all dilated strictures to contract through the preponderance of fibrous tissue in their walls, and stricture of the nasal duct is no exception.

The best means of preventing contracture is to insert a small leaden *style* into the dilated duct and allow it to remain there permanently, removing it every month or two for cleansing purposes. If the style sets up much irritation it may be removed for a fortnight.

Some few cases have proved so troublesome and intractable that recourse has been taken to dissecting out the lachrymal sac.

Obstruction to the flow of tears may be caused by pressure from without when the bony surroundings of the apparatus have been fractured and in healing have thrown out a mass of bony callus, such as occurs after severe blows and gun-shot injuries. Gummatous deposit in the region of the sac and malignant growths and bone necrosis in the nose are also causes.

Summing up, epiphora may be caused by:—1. Partial or total occlusion of the puncta. 2. Catarrhal swelling of the mucous membrane of any portion of the lachrymal tract. 3. Obstruction from within, *i.e. stricture*; (*a*) of canaliculus; (*b*) of nasal duct causing blenorrhœa or dacryocystitis. 4. Obstruction from without due to (*a*) bony growth or necrosis; (*b*) malignant growth; (*c*) tertiary syphilis.

### "The Hospital" Convalescent Fund

THE Hon. Secretary acknowledges, with many thanks, the receipt of 10s. with a note as follows: "A small thank offering from a nurse who has recovered from a serious illness in hospital, and who wishes to remain anonymous." The Hon. Secretary will be glad to forward to A. T., who sends no address, the receipt from Alderman Treloar for 5s. kindly forwarded by her for the Cripples' Dinner Fund.

### Christmas and New Year Festivities in the Hospitals.

BY OUR OWN COMMISSIONERS.

(*Concluded from page* 195.)

### St. George's Hospital.

Christmas was as usual observed very quietly at St. George's Hospital. On Christmas Day tea was provided by kind friends for all the inmates. The wards were tastefully decorated, the two containing children being provided with Christmas trees, which were all laden with toys. There was special fare for all who were able to partake of it. The patients were permitted to receive visits in the afternoon; gifts of books, cards and toys were made to the children, and in the evening the wards were illuminated with fairy lights. On December 29th an entertainment for the patients took place, which consisted of an exhibition of moving pictures and a marionnette performance. Additional interest was excited by the presence of Princess Victoria of Schleswig-Holstein, who distributed the presents to many of the patients, her visit to the wards evidently affording much pleasure to all.

### London Temperance Hospital.

The patients at the London Temperance Hospital spent a very bright and happy Christmas Day. Dinner over, they were entertained in the afternoon with a concert given by friends of the hospital. Tea was at four and each patient was allowed to invite one friend, and yet another for the great event of the day—the entertainment given by the nurses in the evening. On this occasion it took the form of picture songs, one of the most effective being "Where are you Going, My Pretty Maid?" The costumes were from Cox's, who supplied them at a nominal price. After the picture songs followed an exhibition of Mrs. Jarley's waxworks, which created much amusement. The entertainment given by the nurses is always the most popular of the Christmas festivities at the Temperance Hospital. Being of the nature of a family party, with no outsiders save the patients' friends, the nurses throw themselves into the spirit of the thing with a zest that makes the performance an immense success. On the 28th a concert was provided for the patients when a long programme was gone through, the chairman of the hospital, Alderman Strong, presiding over the proceedings.

### Brompton Hospital for Consumption.

The inmates of the Brompton Hospital for Consumption spent a very enjoyable Christmas Day, for besides the usual Christmas fare they were accorded permission to wander over the whole hospital and visit one another's wards. On this day, too, the one day in the year, the men are granted the much-coveted privilege of smoking in their wards. On New Year's Eve the patients, to the number of nearly 300, assembled in the concert hall to witness the distribution of gifts from a Christmas tree. Only 20 patients were unable to be present, not being fit to leave their beds. The tree, which was a very fine one, was the gift of Miss Hedley, and was decorated and equipped under the supervision of Mrs. Price, the lady superintendent. The proceedings were opened by Dr. Hedley in the guise of Father Christmas, who made an amusing speech to the audience, into which he contrived to introduce the names of everyone connected with the hospital. The next event was the stripping of the tree by the resident medical staff, who handed the gifts to the nurses for distribution to their patients. By an ingenious system of marking, each nurse was responsible for certain numbers, say 1 to 40, and so on, so that all confusion was obviated. Every patient in the hospital received a present, those for the adults being of a thoroughly useful character, such as socks, gloves, and comforters for the men, and warm clothing of a like description for the women, while the children obtained a variety of toys. Much amusement was caused by several bladders in the form of monster worms, which the patients took great delight in striking from hand to hand all over the hall. Music was provided during the evening by the Misses Mant.

### The National Hospital.

The patients at the National Hospital for the Paralysed and Epileptic had roast beef and plum pudding for dinner, and all the men able to smoke were given a packet of tobacco. Visitors were allowed to see the patients in the afternoon, and Christmas teas were provided in nearly all the wards. This is the only time in the year when the nurses and the patients are able to have a meal together. The nurses sang carols in the morning and evening. The patients at this hospital are extremely fond of music, and hailed with intense satisfaction the announcement that fortnightly concerts are to be held in the out-patients' hall during January, February, and March.

### London Homœopathic Hospital.

The annual Christmas entertainment to the patients of the London Homœopathic Hospital was held on New Year's Eve. All the wards were prettily decorated, one in particular, Durning Ward, being distinguished by an effective display on a table at the entrance of the ward, consisting of an elegant parterre of pink paper dotted over with tiny coloured candles, surrounded by brighter lights in tulip-shaped fairy lamps. Fairy lamps, indeed, played a leading part in all the decorations. In the tea-room a very pretty effect had been produced by placing the lamps within coloured paper cut to resemble yellow or white water lilies. But the chief centre of attraction was, naturally, the children's ward, where stood a really majestic Christmas tree. Festoons of evergreens formed an archway to the ward, and inside trails of ivy hung down all the walls, while flowers in pots and palms lent beauty to the scene. Fairy lamps were in great profusion here also, hanging in all the windows, enclosed in various coloured paper shades. The tree itself stood at the far end, glittering with long chains of glass ornaments and lighted by electric light in white, yellow, crimson, and green bulbs. From its branches hung numbers of dolls and other light toys, while heavier articles were piled at its foot. Altogether it was a most gorgeous sight and it was small wonder that little Tom who had "never seen a Christmas tree before" gazed with wide eyes at the wonders before him.

### Hospital for Sick Children.

The Christmas festivities at the Hospital for Sick Children, in Bloomsbury, began on Christmas Day, when the medical staff visited the wards and gave an impromptu performance to a highly appreciative audience. But "Tree Day," as the little inmates call the annual Christmas entertainment, took place on December 29th. On that great and wonderful day—which is always looked forward to with so much gleeful anticipation—every ward in the hospital was transformed for the time into a glade of fairyland. A profusion of gay flowers decked the tables, while festoons of ivy and trailing smilax adorned the walls and windows. In every ward there was a stately Christmas tree, some illuminated by electric light, others by numbers of many coloured tapers, all well laden with every

### CHRISTMAS AND NEW YEAR FESTIVITIES IN THE HOSPITALS—*Continued.*

conceivable bonbon and toy, while at the foot of each were piled up heaps of inviting looking parcels. Alice Ward, which contains the "Punch" and "Lewis Carroll" cots, was lighted up entirely by rows of crimson fairy lamps, strung diagonally across the room, which shed a rosy glow over the eager little faces. Kind friends of the hospital had lent a piano to each ward, and these were never allowed to be silent for long. A gramophone did its share towards the general entertainment, whilst an amateur string band paid welcome visits to the various wards. When the Christmas trees were all illuminated came the great moment, and the presents were distributed. Such big parcels they were too, each containing some three or four gifts. "I never thought I should get a doll," said one little maiden in awe-struck tones, as she gazed with delight on a smartly-dressed lady who reposed on the top of her parcel. Beneath the doll was a picture-book, a puzzle, a box of soldiers, and a miniature washstand with jug and basin. Such a wealth of gifts was almost overwhelming, but the small people soon recovered from the first shock of surprise, and proceeded to claim the admiration of doctors, nurses, and visitors for their treasures.

### East London Hospital for Children.

Never before has the number of acceptances for the Christmas entertainment at the East London Hospital for Children been so large, and the out-patients' hall was accordingly well filled on Wednesday the 30th ult., when the little in-patients, brought down and placed on mattresses, evidently enjoyed the clever performance of living marionettes to the full. This was succeeded by the lighting-up of two enormous trees, the gift of Mr. T. Howell-Buxton, laden with toys sent by Royal and other donors, and by the appearance, in scarlet coat, top boots, and holly wreath, of Father Christmas, who was greeted with a chorus of delight and cries of "Here, Doctor!" and "Please, Doctor!" as the work of distribution proceeded. The band of the Royal Horse Guards Blue played at intervals, and shortly before six o'clock the children were carried back to the wards, their arms full of new treasures. The hospital was tastefully decorated, Chinese lanterns and evergreens decked the hall and corridors, and in the wards were chrysanthemums, violets, daffodils, and lilies of the valley, while doorways and pillars were wreathed with trails of ivy. Presents were provided for the nursing staff by the Chairman of the hospital, Colonel Needham, who with Mrs. Needham was present at the entertainment. Among the guests were also Sir H. Samuel, M.P., Lady Samuel, Canon Erskine Clarke, and many local clergy. The convalescents were not forgotten, and a tree, decorations, and toys were subsequently sent to Bognor.

### Mount Vernon Consumption Hospital.

The festivities at the Mount Vernon Consumption Hospital, Hampstead, commenced on Christmas day with the singing of carols by the nurses. The annual Christmas dinner to the patients followed, and after dinner a short concert was given. At tea-time two nurses, dressed up as Father and Mother Christmas, presented each of the patients with a Christmas gift, a work in which they were assisted by two other nurses in fancy dresses to represent the Old Year and the New Year. In the evening the patients had a concert to themselves, and Miss Longstaff, the matron, and the nurses spared no pains to make the day pass as pleasantly as possible. On Monday, December 28, an additional Christmas concert was given by friends of the hospital, after which supper was served to the patients in the hall, cake, coffee, and fruits being dispensed instead of the usual fare. The nurses had a party on the 30th.

### City of London Lying-in Hospital.

"Five o'clock, nurse, and a merry Christmas" were the first words which greeted the nurses on Christmas morning. A few minutes later, amid laughter and bustle, they hurried with their carol book and their candles to the appointed starting place. As the first ward was entered, to the strain of "Christians, awake," it was easy, notwithstanding the semi-darkness, to note the patients' eager, gratified faces as they discovered on the beds their Christmas gifts of warm clothing for themselves and their babies. After the carols had been sung in every ward, the patients had their breakfast, another little mite meanwhile being superintended into this world, and then the nurses found time for church. Next came the grand preparation for dinner. As many of the patients as were able were dressed and brought into one ward; even the babies wore their best smile, together with long white robes and pink ribbons. The enormous table, decorated with flowers, ferns, holly, oranges, and crackers, was prepared, and then, at twelve o'clock, dinner was served. A truly joyous sight! All the poor women thoroughly enjoyed for once a real good dinner, their happy faces seeming to say, "Baby or no baby, feed well I will." After a rest there was a little entertainment of music and singing, which they all much appreciated, the babies chiming in whenever they thought it necessary, and so the tea hour came. At the matron's wish, the nurses sang another carol before the babies and their respective mothers had their faces and hands washed and were put to bed, after what they termed "a real good Christmas." At eight o'clock the nurses went into their dining hall and had dancing and games till close upon one o'clock.

### St. Marylebone Infirmary.

By invitation of the guardians a large number of people visited St. Marylebone Infirmary, North Kensington, on New Year's Eve to inspect the wards. Great pains had been taken to make everything look bright and Christmas-like, and not only the medical and nursing staff but the patients had entered into the spirit of it, with admirable result. Chinese lanterns and wreaths of evergreen, relieved by pretty paper lamp shades and gay flags, decorated wards and corridors, and the waxworks, which were placed in unexpected corners, were quite a novel feature. One of these, on the male side, represented a doctor who might have been Esculapius himself but for the label, "Asepsis," which was thrown across his breast; he was engaged in catching with a butterfly net sundry evil-looking parasites and microbes which were suspended from evergreens over his head, while a neighbouring wall bore a legend in which the following advice was given: "In order to be entirely healthy, eat nothing, drink nothing, smoke nothing, and even before breathing make sure that the air has been properly sterilized." Another clever figure, the work of a bedridden patient, was a gipsy woman selling clothes pegs, and standing by a fire of twigs and fairy lamps, over which hung an iron crock. "I want to see Dr. Lunn," was inscribed on a letter she held in her hand. A man-of-war in a corridor was the work of another patient, while a blind man had with great patience produced some large leaden cannon balls, by rolling together the linings of packets of cocoa. On the female side was a figure of Father Christmas, his scarlet coat and umbrella sprinkled with snowflakes. Some very good paper Chinamen and rabbits, and a reproduction of Wee Macgregor were the work of a probationer. Palms, flowers, dainty hanging lamps, and mottoes helped the general effect. Carols were sung in the wards by the nurses, who were assisted by members of the medical staff, and the officials present included the medical superintendent, Dr. J. R. Lunn, the matron, Miss Grace Ramsden, and the chaplains.

MILE END INFIRMARY.

Christmas Day was ushered in at Mile End Infirmary in the early morning by many of the nursing staff singing carols at the entrance to the various wards, and later on by the Guardians' School Band playing selections of music between the pavilions. The wards were visited by several guardians and friends, and all expressed their great pleasure in seeing the interest that had been taken by the nurses in making their wards bright and cheerful with the decorations. The patients, numbering 500, were delighted with the good things provided for them by the guardians. In the afternoon a large Christmas-tree well laden with all sorts of useful toys for the children was exhibited. The toys were distributed, also a new sixpence to each child, kindly sent by the editor of *Truth*. In the evening the nursing staff gave an excellent variety entertainment to the patients, which was much enjoyed by all.

NORTH-EASTERN HOSPITAL FOR CHILDREN.

On Thursday afternoon last nearly a hundred and fifty children were entertained in the out-patient hall at the North-Eastern Hospital for Children, Hackney Road, Bethnal Green. Their ages varied from four to twelve years, and they all came from the immediate neighbourhood. A very interesting and pleasing feature is the fact that the whole of the cost was defrayed by the efforts of the nursing staff who collected the necessary money, under the superintendence of Miss Copman, out-patient sister, who was responsible for the arrangements. After tea there was a magic-lantern show in the hall, and several useful gifts were made to each little guest from a large Christmas tree, in addition to toys and cake. A considerable number of children took part in a cake-walk, which caused much amusement, a prize being awarded to the most successful.

WAKEFIELD UNION INFIRMARY.

Christmas Day was spent very happily at the Union Infirmary, Wakefield, both by the patients and staff. At 6 A.M. the nurses visited each ward and sang carols, this being much appreciated. The usual English Christmas dinner was served at 1 P.M., after which coffee was handed round, fruit distributed, and tobacco and pipes for all who wished. During the day several members and friends of the committee visited the infirmary, including the Mayor and Mayoress of Wakefield who distributed toys to the children. During the evening the Nurses' Troupe of Pierrots, under the management of Sister Milnes, gave an excellent entertainment in the wards, consisting of songs, part-songs, 'cello solos, pianoforte solos, duets, and humorous readings. The selections by the "Tommy Talker" Band created roars of laughter, fairly bringing down the house. Miss Lightowler, the superintendent, acted as chairman, and a most enjoyable day was brought to a close by a ringing cheer for the nurses. During the day the nurses presented to Miss Lightowler a case of silver teaspoons as a token of regard.

PORTSMOUTH INFIRMARY.

AT Portsmouth Workhouse Infirmary Christmas was kept up in good old-fashioned style. The wards were beautifully decorated, the patients were as trim as they could be made, and the fare for Christmas dinner, viz., roast beef, plum pudding with sauce, fruit and sweets, also tobacco for the men and tea, etc., for the women, besides beer and lemonade to drink, was much appreciated by those who were allowed to partake of it. The probationers also got up an entertainment of songs and recitations, as well as two capital farces, which were repeated in different parts of the building, and the whole went off well. There was a Christmas Tree for the children and many nice toys for the sick little ones were sent from the Portsmouth League of Love.

WEST CORNWALL MINERS' HOSPITAL, REDRUTH.

Patients of the West Cornwall Miners' Women's and Children's Hospital at Redruth as usual have had this year a most enjoyable time. Commencing early in the week with an entertainment of music, a farce, and a gramophone, festive pleasures were continued on Christmas Eve. First there was the nurses' "At Home," and later in the evening the children's Christmas tree, gaily decorated with pretty dolls and other toys, oranges, apples, and fruit. Some excellent music, vocal and instrumental, was well given by one friend of the hospital, and a large number of useful and pretty articles of clothing, etc., were distributed to the patients by another. Appropriate characteristic mottoes for the doctors caused much amusement. The presents for the patients were provided by contributions collected by matron and nurses from hospital committee, medical, nursing, and house staff, outside friends, and some of the workers at the mines, the tree and many toys being also gifts. Upwards of fifty friends attended the entertainments. The wards were prettily decorated in holly, evergreens, and red, white, and blue. On Christmas Day the usual liberal seasonable fare was greatly appreciated, geese and turkeys being sent as before by a friend. The matron, nursing and house staff deserve credit for the admirable manner in which everything possible was arranged for general comfort.

PRUDHOE CONVALESCENT HOME, WHITLEY BAY.

THE patients at the Convalescent Home, Whitley Bay, who are chiefly miners, say that they never spent such a Christmas in their lives. The spacious and lofty dining-hall, which seats 170, was tastefully decorated with evergreens, chrysanthemums and ferns, and the usual mottoes. But the centre of attraction was a magnificent Christmas-tree, laden with useful gifts—socks and knives and pipes for the men, and shawls or scissors for the women. It was also hung with oranges, apples, crackers, and pretty little bags of sweets. Dinner was served at 1 o'clock, and consisted of turkey and roast beef, followed by plum-pudding. This was greatly enjoyed by all, together with the apples, oranges, and nuts provided for desert. Tobacco was also given to each man to enjoy in the smoking-room afterwards. The Christmas-tree was lighted up at 6 P.M., while the patients watched it. Carols and songs were sung. The tree, and everything on it, were gifts. Some friends sent money to the matron to buy what was suitable for the patients. Others forwarded their contributions all ready to be labelled and hung up. At 8 o'clock supper was served, after which the centre of the hall was cleared and games were played, in which all joined excepting those with fractured legs and the worst cases of rheumatism. The fun was kept up until 10 P.M.

### Presentations.

ST. OLAVE'S SCHOOLS, SUTTON.—A pleasing ceremony took place last month when Miss F. E. Capes, the matron of St. Olave's temporary schools, at Sutton, was presented by the officers and staff with a handsome framed photograph of the children and staff, entitled "The Last of the Sutton Schools," and a gold bracelet.

BROOKLYN INSTITUTE, ANERLEY.—On Christmas Day the nurses of the Brooklyn Institute, Anerley Road, S.E., presented the matron, Mrs. Cameron, and Miss Aubert with a set of fish knives and forks and a bread fork.

### Queen Victoria's Jubilee Institute for Nurses.

Her Majesty Queen Alexandra has been graciously pleased to approve the appointment of the following as "Queen's Nurses" to date January 1st, 1904:—

ENGLAND.

| Nurse. | District Training at | Working at |
|---|---|---|
| Annette Clara Durrell Lamb | Bacup | Somercotes |
| Ada Louisa Davison | Battersea | Crawley, Sussex C.N.A. |
| Emily Smith | „ | Stafford |
| Helen Florence Barry | Bermondsey | Darlington |
| Millicent Fanny Prior | „ | New Mills |
| Evelyn Ogilvy | „ | St. Austell |
| Isabella Griffiths | Birkenhead | Birkenhead |
| Elizabeth Goodlet | „ | „ |
| Ada Constance Hallam | Birmingham (Moseley Rd.) | Birmingham |
| Maria Keywood | Birmingham (Newhall St.) | Warsop |
| Annie Wotherspoon | Bloomsbury | Consett |
| Lucretia Hill | „ | Aylesbury |
| Ethel Elizabeth Bills | „ | Upholland |
| Alice Gunner | „ | Truro |
| Annie Susan Hiscock | „ | Chatham |
| Margaret Milne | „ | Hackney |
| Ruth Elizabeth Randall | „ | Silvertown |
| Edith Redmile | „ | Bloomsbury |
| Rose Sweetlove | „ | Hayes |
| Winifred Starr | Bolton | Bolton |
| Lilian Alice Jones | „ | „ |
| Elizabeth Marian Dunn | Brighton | Sheffield |
| Annie Wood | „ | Gloucester |
| Sarah Sophia Wood | „ | „ |
| Alice Maud Koe | Brighton | Brighton |
| Emma Clark | Burnley | Burnley |
| Susannah Settle | „ | „ |
| Elizabeth Porteus | Cardiff | Bacup |
| Margaret Louisa Cowley | Carlisle | Carlisle |
| Clara Orpin | Chelsea | Silvertown |
| Edith Corrie | „ | Chelsea |
| Alice Petty | Derby C.N.A. | Swanich |
| Fanny Fairbrother | Dublin (St. Patrick's) | Darwen |
| Agnes Elsie Cadbury | E. London | E. London |
| Jessie Bond Chambers | Gateshead | Southampton |
| Ethel Mary Heaster | Gloucester | Gloucester |
| Annie Macmillan | „ | „ |
| Ellen Howell | Hammersmith | Ditchingham |
| Ada Beatrice Barrow | Hants C.N.A. | Headley |
| Emma Sarah Heal | Haydock | Haydock |
| Sara McCready | Hull | Hull |
| Julie Japing | Kensington | Kensington |
| Louisa Esther Randall | Lincs. C.N.A. | Caistor |
| Grace Evangeline Gomm | „ | Moulton |
| Elizabeth Farquharson | Liverpool (Centr. Home) | Liverpool |
| Jane Elizabeth Wynne Puffitt | „ | „ |
| Minnie Starbuck | Liverpool (Kirkdale Rd.) | Liverpool, Kirkdale Road |
| Alice Maud Rigby | „ | „ |
| Catherine Gourlay Bartlett | „ | „ |
| Alice Sylvia Barton | „ | „ |
| Fanny Walmsley | Liverpool (Overton St.) | Sheffield |
| Mary MacKinlay Weir | „ | Kimpton |
| Annie Mary Barlow | Liverpool (Shaw St.) | Liverpool, Shaw Street |
| Martha Blanche Edwards | „ | Hebden Bridge |
| Fanny Cripps | Manchester (Ardwick Grn.) | Manchester, Ardwick Green |
| Sarah Jones | „ | „ |
| Anne Sowerbutts | Manchester (Brad'ord Home) | Manchester, Bradford Home |
| Henrietta Tyson | „ | „ |
| Margaret Rogerson | Manchester (Harpurhey) | Manchester, Harpurhey |
| Mary Elizabeth Barlow | Manchester (Hulme H.) | Manchester, Hulme H. |
| Adelaide Lilias Wimberley | Notts C. N. A. | Burton Joyce |
| Adella Catherine Margaret Lowman | Portsmouth | Newmarket |
| Sarah Footner | „ | Helston |
| Mary Eliza Clarke | „ | Portsmouth |
| Elizabeth Winifred Higgins | „ | Faringdon |
| Lilian Fisher | Portsmouth (and Salford) | Liverpool, Central Home |
| Mabel Sophia Hewitt | St. Neots | St. Neots |
| Lilian Taylor | Salford | Norton |
| Mary Elizabeth Davis | Shoreditch | Shoreditch |
| Mary Anne Holbrook | „ | Widnes |
| Ethyl Jackson | „ | „ |
| Mary Moore | „ | Shoreditch |
| Sarah Ellen Morris | „ | „ |
| Margaret Wheatcroft | „ | Bacup |
| Ada Jane Giles | Sunderland | Sunderland |
| Elizabeth Briggs | Torquay | Sholing |
| Catherine Sarah Rees | Walworth | Barrow |
| Maria Edis Rolls | „ | Sheffield |
| Florence Steele | Westminster | Westminster |
| Gertrude Petty | Windsor | Windsor |

WALES.

| Nurse. | District Training at | Working at |
|---|---|---|
| Emma Griffiths | Camberwell | Machynlleth |
| Minnie Anscombe | „ | Pembroke Dock |
| Ellen Selina Myers | Cardiff | Cardiff |
| Elizabeth Rachael Bailey | „ | Colwyn Bay |
| Evelyn Windsor | „ | Ruthin |
| Elizabeth Jane Jones | Salford | Harlech |
| Sarah Bridge | Shoreditch | Williamston |

SCOTLAND.

| Nurse. | District Training at | Working at |
|---|---|---|
| Christina Duff Bailie | Edinburgh | Blantyre |
| Agnes Reid | „ | Crieff |
| Janet Arnott | „ | Edinburgh |
| Ann Ferguson | „ | „ |
| Martha Jane Haigh | „ | „ |
| Agnes Lorimer Howat | „ | „ |
| Margaret S. Milne | „ | „ |
| Elizabeth Jane Rose | „ | „ |
| Elizabeth Towns | „ | „ |
| Louisa Jane Wilson | „ | „ |
| Lucy Violet Watchorn | „ | Elgin |
| Elizabeth Munro | „ | „ |
| Ethel May Payne | „ | Fair Island (Shet'and) |
| Annie Hartley | „ | Galashiels |
| Kate Strang | „ | Kelty |
| Rosina Matthews McArthur | „ | Lerwick |
| Margaret Bell | „ | Lochgilphear |
| Florence Adams | „ | Tollcross |
| Minnie Criddle | Glasgow | Glasgow |
| Isabella Melville | Paisley | Paisley |

IRELAND.

| Nurse. | District Training at | Working at |
|---|---|---|
| Elizabeth F. Cusack | Dublin, St. Lawrence's | Balladangen |
| Catherine Mary Wills | „ „ | Carna |
| Mary Boyle | „ „ | Dublin |
| Anna Jane McGrath | „ „ | „ |
| Bridgid Deery | „ „ | „ |
| Gertrude Evelyn Grace Chapman | „ „ | „ |
| Margaret Moran | „ „ | Kilcommon, Erris |
| Margaret Farrelly | „ „ | Killorglin |
| Olive Katharine Gawley | Dublin, St. Patrick's | Dublin |
| Harriet Fleming | „ „ | Newtownards |
| Maud Maria Ruttledge | „ „ | Portadown |
| Letitia Charlotte Scott | Londonderry | Londonderry |

### Appointments.

Charing Cross Hospital.—Miss Ethel Kaberry has been appointed out-patient sister. She was trained at the Bristol General Hospital, and has since been sister at the East Suffolk Hospital, Ipswich, and sister of the men's corridor at Mount Vernon Hospital, Hampstead, N.W.

Faringdon District Nursing Association. — Miss Winifred Higgins has been appointed Queen's nurse. She was trained at the Steyning Infirmary, Shoreham, Sussex. She holds the L.O.S. certificate.

Kilkee Union.—Miss Annie MacSweeney has been appointed assistant nurse. She was trained for two years by the Meath Workhouse Nursing Association at the Children's Hospital, Temple Street, Dublin.

Maidenhead Union Infirmary.—Miss Florence Grey has been appointed on probation assistant nurse. She was trained for two years by the Meath Workhouse Nursing Association at St. Peter's Home, Kilburn.

Reigate and Redhill Cottage Hospital.—Miss Agnes Elizabeth Taylor has been appointed matron. She was trained at St. Thomas's Hospital, London, and has since been assistant night superintendent, sister of medical ward, and sister of children's ward, in the same institution.

West Norfolk and Lynn Hospital.—Miss Kathleen Smith has been appointed day and theatre sister. She was trained at the Essex and Colchester General Hospital and at the Royal Hants County Hospital. She has since been sister at the Essex and Colchester Hospital; sister at the Shirley Warren Infirmary, Southampton; and charge nurse at the Victoria Isolation Hospital, Winchester.

### Echoes from the Outside World.

### Royal Visit to Chatsworth.

AFTER spending the opening days of the New Year quietly at Sandringham, the King and Queen left on Monday in order to visit the Duke and Duchess of Devonshire at Chatsworth. Their Majesties, accompanied by Princess Victoria, reached Rowsley at five o'clock in the afternoon, a guard of honour consisting of 95 men of the 2nd Volunteer Battalion of the Derbyshire Regiment being drawn up at the elaborately-decorated station. There was a short wait in the reception-room, during which the King expressed to Sir Ernest Paget his satisfaction with the arrangements made on the Midland Railway. On their way to Chatsworth the Royal visitors were met by a large party of torch bearers. As the procession approached the Derwent Bridge the sky suddenly became illuminated by rockets from the terrace, by brilliant searchlights, and by the ruddy glow of coloured fires. Prominent among the devices was a crown between letters representing their Majesties' initials, and the word "Welcome." On Tuesday morning the King went out shooting, and later in the day the party were joined by the Queen.

### Death of Princess Mathilde.

A VERY remarkable and illustrious woman died on Sunday evening at her residence in Paris. Princess Mathilde Létitia Wilhelmina Bonaparte, who was 83, last summer caught her foot in a carpet and fell heavily to the ground, breaking her thigh. Owing to her advanced age she never entirely recovered from the shock. She was the daughter of Jérôme Bonaparte, King of Westphalia, who married the Princess Catherine of Würtemberg. She was descended from George II. of England, from Frederick William I. of Prussia, from the Saxon Emperors, from the Royal lines of Spain, Portugal, Denmark, and Norway, from St. Louis and many subsequent kings of France. She was third cousin to King Edward, second cousin to Czar Alexander II., and fourth cousin to Kaiser Frederic III., and when she was born she was within measurable distance of the succession to the Throne of Great Britain. As it was, the only throne she occupied was that of the intellectual life of Paris. Possessing not only all possible charms of manner, she was also exceedingly accomplished, and herself frequently exhibited at the Salon. She established the Asila Mathilde for incurable girls and charitable works occupied a great deal of her attention. The Princess was the last of the Bonapartes who were living at the time of the great Emperor's death. As recently as December 2, when she was conscious of her approaching end, she caused her chair to be carried to the window of her apartment, and, drawing the attention of those around her to the radiant sunshine, exclaimed, with a happy smile, "It is the sun of Austerlitz."

### Homes for Officers' Widows and Daughters.

IT will be remembered that in 1899 a scheme for providing homes for the widows and daughters of officers was started with subscriptions raised privately, and four of the widows and daughters of poor officers of the rank of general, two of colonel, two of commander, and four of captain, were temporarily provided with suites of self-contained apartments in Chelsea, rent free. An extension is now in process with a view of placing the scheme on a permanent footing. This is mainly due to the personal interest and support of Queen Alexandra, who has devoted to this object the Coronation gift of £10,000 presented to her Majesty by an Australian gentleman now resident in England, and also £5,000 from her Majesty's War Fund. Twenty-four suites of apartments are now in course of construction at Wimbledon Park, on a site of 3½ acres, and thirty-six more will be erected as soon as funds are forthcoming. Although the candidates are limited to ladies whose incomes do not exceed £100 per annum, and who are not less than fifty years of age, no fewer than sixty-seven qualified under these conditions.

### Great Fire at Chicago.

DURING the progress of the pantomime, *Bluebeard Junior*, at the newly-finished theatre, the Iroquois, in Chicago, on Wednesday afternoon last week, a fire occurred on the stage which proved ultimately to be equal in horror to the Ring disaster in Vienna. The cry of "Fire" at once created a panic, in which many were crushed. The audience made a wild rush for the doors with the result that they were speedily blocked, and it is stated that in many cases the fire escapes of the theatre had not been completed, and therefore were of no avail. The fireproof curtain, notwithstanding valiant efforts to lower it, stuck midway, and by creating a draught fanned the flames. Nearly all the actors escaped as a result of the bravery of the leading comedian, Mr. Eddie Foy; many of the chorus girls, who were imprisoned in the uppermost gallery behind the scenes, were rescued through the courage of the boy in charge of the lift, who ran his "elevator" up in spite of the fire and brought them down uninjured; a stage hand saved a dozen women by making them join hands and thus ensuring that they followed him through a coal-hole into the open air; and Bishop Muldoon, who was passing the theatre at the time the fire broke out, at once entered the building and gave his absolution to the dying. He also helped to hand out those who were injured only. In the end about 700 people perished, many being suffocated by the escaping gas as they sat in their seats. The temperature was below zero at the time, and even those who got into the street were of course without wraps, and suffered much from the cold before they could procure shelter. Touching messages of sympathy with the sufferers in the disaster from King Edward and Queen Alexandra and the Prince and Princess of Wales have been gratefully acknowledged by the President of the United States.

### Old Masters at Burlington House.

THE most striking feature of the exhibition of the Old Masters, opened at Burlington House on Monday, is its exceeding variety. The first gallery is almost entirely occupied by the works of the Italian masters, and the eye will rest with pleasure upon a splendid Dürer, a portrait of the painter's father. The second gallery is devoted to the painting of Sir Thomas Laurence, the once celebrated President of the Academy, who, after being held in high repute, fell into disfavour, and has only again risen into esteem the last ten or fifteen years. His paintings are very unequal. Amongst those specially to be noticed as possessing much beauty are "Miss Farren, Countess of Derby," "Master Lambton," son of the Earl of Durham, and the "Countess of Leitrim and Daughter." Considerable interest also attaches to the same artist's portrait of Pius VII.—the Pope who crowned Napoleon and who was afterwards imprisoned by him at Fontainbleau—with his slight, weak figure and his worn countenance. There is one Rembrandt, a portrait of a lady; one very good Hobbema, "On the Road to Scheveningen;" a picture of Verona by Canaletto, and a fine Raeburn. Amongst Mr. Horsley's works "Mary, Queen of Scots in Captivity" is, perhaps, his best example; whilst "Cattle—Evening," by Mr. Sidney Cooper, painted in 1850, looks as fresh in colour as if the canvas had just left the painter's hand. The Italian bronzes, mostly lent by Mr. Pierpont Morgan, Mr. Wernher, Mr. Beit, and Mr. Salting are a fine collection.

### Notes and Queries.

**FOR REGULATIONS SEE PAGE 135.**

*Home.*

(126) Can you tell me of a cripples' home which would take a crippled man, aged 28?—*B. C.*

He is too old for a cripples' home. Perhaps a home for incurables might meet the case.

*District Nurse.*

(127) I should like to be a district nurse. Please tell me where I could write to in Edinburgh for particulars.—*M. P. T.*

The Hon. Secretary, Qeeen Victoria's Jubilee Institute for Nurses, Scottish Branch, 2 Lansdown Crescent, Edinburgh.

*Abroad.*

(128) Can you inform me of the addresses of the nursing homes at Rome, Florence, Nice, and Cannes, and also how best to seek engagements at any of these places?—*Traveller and Marie O.*

The Anglo-American Nursing Home, Rome, the Association of Trained Nurses and Masseuses, 7 Via Rondinelli, Florence; the Nice Nursing Institute, Villa Pilatte, Avenue Desambrois, Nice; there is none at Cannes, but the British Hospital, Sunny Bank, Petit-Juas, Cannes, employs English nurses. Apply as usual. So does the English Nurses' Institute, Casa Roggeroni, Sunny Bank, San Remo.

*South Africa.*

(129) I shall be greatly obliged if you can furnish me with the addresses of nursing institutions in South Africa as I wish to settle there, or if you can let me know where to write for information.—*A. J. A.*

You will obtain reliable information from the South African Expansion Emigration Committee, 47 Victoria Street, Westminster, S.W.

*Weir-Mitchell. Lunacy Laws.*

(130) 1. What is the Weir-Mitchell treatment? 2. Is is quite correct for a medical man or a private family to have charge of mental patients without a license? etc., etc.—*Mona.*

1. You will find a full explanation of the Weir-Mitchell treatment in "Nursing: its Theory and Practice," by Percy G. Lewis, M.D., M.R.C.S., L.S.A., price 3s. 6d., which can be obtained from the Scientific Press. 2. See the articles which appeared in THE HOSPITAL for February 21st, March 28th, and April 18th, 1903, entitled "The Law Relating to Lunatics and Persons of Unsound Mind."

*Hospital Training.*

(131) I am 21. I want general training in a good hospital. Would it be wise for me to go to a children's hospital for two years, or would fever training hinder my obtaining further training in a large hospital?—*L. S. T.*

Training in a children's hospital before general training might be useful to you.

*Maternity.*

(132) Will you kindly tell me if it is correct when engaging to attend a maternity case to count the "month" from the first of one month to the first of the next, or four weeks.—*Nurse G.*

It is usual to reckon four weeks.

*Nurses' Hostel.*

(133) Will you tell me if there is a nurses' hostel in Dublin or Killarney. If not where could two nurses get board and lodging at moderate terms?—*C. J.*

There is no nurses' hostel in Dublin, but doubtless you could obtain suitable accommodation through La Touche G. F. S. Lodge, 13 South Frederick Street, Dublin.

*Deafness.*

(134) Will you kindly say if the Drouet Institute is a bogus or a genuine institute?—*Ouida.*

It is not recognised by the medical profession.

**Important Nursing Textbooks.**

"The Nursing Profession: How and where to Train." 2s. net; 2s. 4d. post free.

"A Handbook for Nurses." By Dr. J. K. Watson. 5s. net; 5s. 4d. post free.

"Practical Guide to Surgical Bandaging and Dressings." By Wm. Johnson Smith, F.R.C.S. 2s. post free.

"The Nurses' Dictionary of Medical Terms and Nursing Treatment." By Honnor Morten. 2s. post free.

"The Human Body: its Personal Hygiene and Practical Physiology." By B. P. Colton. 5s. post free.

"Art of Feeding the Invalid." (Popular Edition). 1s. 6d. post free.

"On Preparation for Operation in Private Houses. By Stanmore Bishop, F.R.C.S. 6d. post free

### For Reading to the Sick.

EPIPHANYTIDE.

WHAT star is this, with beams so bright,
More beauteous than the noonday light?
It shines to herald forth the King,
And Gentiles to his cradle bring.
See now fulfill'd what God decreed,
"From Jacob shall a star proceed;"
And Eastern sages with amaze
Upon the wondrous vision gaze.
The guiding star above is bright;
Within them shines a clearer light,
Which leads them on with power benign
To seek the Giver of the sign.

*From the Latin.*

The Epiphany is the greatest of the three Christmas festivals. It is the first public acknowledgment of our Lord as King by the Gentile world.

Turn your eyes to the Three Kings as they kneel before the divine Babe at Bethlehem. What have they brought? Have they brought (as they easily might have done) a few thousand sheep or camels, or works of art—in a word, gifts that *show*? No, their gifts are concealed in their robes or carried in their hands; they are of small compass, with no show about them, but "precious in the sight of God." "And *opening* their treasures, they offered Him gifts: gold, frankincense and myrrh." These are accepted by our Lord; they are the only gifts He ever accepted, and the sick can bring them equally with the sound, nay, in greater measure. The gold of innocence of life, in which all is done as God wills it and because God wills it; the frankincense of humble prayer, our every act, our sighs and tears, our pains and hours of gloom, our acts of obedience, offered to God as prayer that rises in a cloud of sweetest incense before His throne; the myrrh of our glad conformity to God's will, meekly accepting what comes day by day in the spirit of the Cross, thus putting self to death and living only for God.

Such is the earthly pilgrimage of all; and the sick can tread the way as well as others, their hands as full of gifts for their infant King as those of others. Let them plod on cheerfully and trustfully. Every day they shall find the Child with Mary its mother, till they be ready to ascend the mount of God, where they shall see that Child "transfigured before them, His face shining as the sun, His garments white as snow."—*Anon.*

Holy Jesus, every day
Keep us in the narrow way;
And, when earthly things are past,
Bring our ransom'd souls at last
Where they need no star to guide,
Where no clouds Thy glory hide.

In the Heav'nly country bright
Need they no created light;
Thou its Light, its Joy, its Crown,
Thou its Sun which goes not down;
There for ever may we sing
Alleluias to our King.

*W. C. Dix.*

